# A Case of Lienteric Diarrhea

**Published:** 1849-10

**Authors:** M. H. Fee

**Affiliations:** Hiltonsville, Indiana


Art. III.—*A Case of Lienteric Diarrhea.* By M. H. Fee, M. D., of Hiltonsville, Indiana.

The case was a young man, aged 17 or 18; a hard working and hearty young man, though of a scrofulous predisposition. His mother, whose physical conformation was the prototype of the son's, died of tubercular phthisis. Every domestic astringent had been exhausted before I was called, but without effect. A physician had been consulted, who had prescribed blue mass, in conjunction with one of the popular nostrums, called "Cholera Mixture," but with no better success.

The physical symptoms were: pulse 125 per minute, small and quick; respiration 26; profuse perspiration; tongue broad, flabby, coated white, tremulous, protruded with difficulty, point and edges of a vivid red, and papillæ enlarged; heart sounds heard at every point of the chest, but normal; respiratory murmur rather puerile, indicating, I think, slight tubercular engorgement; percussion gave a rather dull sound; sleepy expression of countenance; no tenderness of the bowels; about twelve alvine dejections per diem, thin, yellow, with lumps of undigested food; food taken into his stomach was found in half an hour in his stools.

I prescribed twenty-six grains calomel, one and a half grs. opium and three grs. sugar of lead. This held the bowels in check twelve hours. It then moved off, bringing dark bile, but the disease went on afterwards unchanged. I prescribed three grains nit. argent. with sufficient bread crumbs, divided into nine pills, one to be taken every three hours, until all were taken. After which, till my next visit, a pill containing two drops creosote, every three hours.

At my next visit, found him with dry, red, square, clean, pointed tongue. The enlarged papilla had disappeared. Some abdominal tenderness below umbilicus. Bowels much less moved. Applied a large blister over the bowels, and a small one on each ankle; gave one and a half grains opium, ten grains calomel and five grains blue mass; directed to let it lie on the bowels till signs of ptyalism appeared; and ordered slippery elm bark drink.

At my next visit, found the dose prescribed at my last visit had lain twenty-eight hours; slight soreness of gums; tongue clean, moist and natural; bowels acting naturally; appetite good; light food remaining as long as in health; alvine dejections natural; digestion good. He is now well,

My last visit was made the 21st ult.

The disease had been running four weeks when I was called to treat it.

Hiltonsville, Ia., Sept. 5, 1849.

